# Rearing of *Rhipicephalus annulatus* ticks on rabbits for the biological transmission of *Anaplasma marginale*

**DOI:** 10.14202/vetworld.2024.903-910

**Published:** 2024-04-25

**Authors:** Sikandar Ali, Abdullah Saghir Ahmad, Kamran Ashraf, Jawaria Ali Khan, Muhammad Imran Rashid

**Affiliations:** 1 Department of Parasitology, University of Veterinary and Animal Sciences, Lahore, Pakistan; 2 Department of Parasitology, Faculty of Veterinary Science, Cholistan University of Veterinary and Animal Sciences, Bahawalpur 63100, Pakistan; 3 Department of Clinical Medicine and Surgery, Faculty of Veterinary Science, The University of Veterinary and Animal Sciences, Lahore 54000, Pakistan

**Keywords:** *Anaplasma marginale*, horizontal, rabbits, *Rhipicephalus annulatus*, transovarial, transstadial

## Abstract

**Background and Aim:**

*Anaplasma marginale* is an obligate intraerythrocytic rickettsial parasite that infects cattle in tropical and subtropical regions. There is no evidence that *A. marginale* inoculation can be used to culture *Rhipicephalus annulatus* in rabbits. This study aimed to determine the molting of *R. annulatu*s larvae, nymphs, and adults on rabbits as well as nymphs and adults of *R. annulatus* on calves with or without *A. marginale*. Transstadial, horizontal, and transovarial transmissions of *A. marginale* in *R. annulatus* reared on rabbits and calves were evaluated.

**Materials and Methods:**

Engorged female ticks were collected from field samples of *A. marginale*-infected and non-infected cattle. We divided the eight rabbits into two groups: A and B. Group A rabbits were infected with *A. marginale* through parenteral inoculation, whereas Group B rabbits were kept as a control. The “clean rabbits” in Group B were observed for tick rearing without *A. marginale*. Polymerase chain reaction was used to screen *A. marginale* in rabbits and stages of tick. The complete life cycle of *R. annulatus* with or without *A. marginale* was observed on rabbits.

**Results:**

A 6.5-day longer life cycle was observed in ticks harboring *A. marginale* than in ticks without *A. marginale*. To observe transstadial transmission, transstadial, horizontal, and transovarial transmissions of *A. marginale* in *R. annulatus* ticks were experimentally observed in one clean calf fed separately with infected nymphs and female adult ticks.

**Conclusion:**

We experimentally observed transovarian, transstadial, and transovarial transmission of *A. marginale* in *R. annulatus* ticks as a biological vector reared on calves and rabbits. We used rabbits as a model animal for rearing *R. annulatus* ticks and culture of *A. marginale*.

## Introduction

*Anaplasma marginale* is a rickettsial parasite that causes anaplasmosis by infecting erythrocytes of cattle. This disease has high mortality and morbidity rates and causes significant economic losses worldwide [1]. Blood-sucking arthropods, such as ticks and flies, can mechanically and biologically transfer *A. marginale* to cattle [2]. Ticks are known to play an important role in the transmission of *A. marginale* [3]. Twenty tick species belonging to five genera (*Rhipicephalus*, *Ixodes*, *Dermacentor*, *Hyalomma*, and *Amblyomma*) have been identified as potential vectors of *A. marginale* [4]. Although *A. marginale* can be transplacentally transmitted, this mode of transmission is not thought to have a significant impact on its epidemiology [5]. *Rhipicephalus annulatus* is a one-host tick involved in the biological transmission of *A. marginale* [6]. *R. annulatus* causes direct economic losses associated with blood feeding during infestation and indirect effects due to the transmission of hemoparasites such as *Babesia bovis*, *Babesia bigemina*, and *A. marginale* [7].

Parasites can be transmitted through ticks through intrastadial, transstadial, transovarial, or vertical or horizontal transmission [8]. Transstadial and intrastadial transmissions of *A. marginale* by *Rhipicephalus microplus* have already been demonstrated [9–12]. *A. marginale* is transovarially transmitted by *R. microplus* to susceptible steers and calves in Mexico [13]. Some studies on transstadial and vertical transmission have suggested that the vectorial competence of ticks to transmit *A. marginale* may depend on the *A. marginale* isolate [14, 15], which has not been evaluated for transovarial transmission. In Argentina, the pathogenic isolate (SIP) of *A. marginale* is transstadially transmitted by *R. microplus* and *Amblyomma neumanni* ticks [9, 16]. Transovarial transmission is an important mode of transmission for many tick-borne pathogens, including several species of *Babesia*, which are well known for their ability to undergo such transmission [17]. *A. marginale* is horizontally transmitted by ixodid ticks, including *Rhipicephalus* spp. and *Dermacentor* spp. *R. microplus* is considered the most important biological vector in tropical and subtropical regions of the world [18].

Various animal models have been used to explore the vertical transmission of *Anaplasma* via *Rhipicephalus* ticks. One such model used steers (neutered male cattle) to examine vertical transmission of *A. marginale* through *R. microplus* ticks [13]. Cattle calves have also been employed as an animal model to investigate vertical transmission of *A. marginale* via *R. microplus* ticks [19]. Molecular techniques, such as polymerase chain reaction (PCR), have the capability to detect pathogens in ticks at all stages of development [19]. Molecular detection of pathogens such as *Babesia* spp. [20], *Anaplasma* spp. [20], and *Rickettsia* spp. [21] in eggs and unfed larvae has been reported. However, only a few reports on the molecular detection of pathogens in transovarial transmission in India have been published [18].

The aim of this study was to use rabbits as a model animal for culturing *R. annulatus* in the presence or absence of *A. marginale* and to observe its transstadial, transovarial, and horizontal transmissions.

## Materials and Methods

### Ethical approval

All experimental procedures were performed according to the guidelines approved by the Ethical Review Committee of the University of Veterinary and Animal Sciences (UVAS), Lahore (No. DR/420, Dated: October 13, 2021).

### Study period and location

This study was conducted from February to June 2022 in the Parasitology Department, University of Veterinary and Animal Sciences, Lahore, Pakistan.

### Collection of ticks

**Figure-1:**
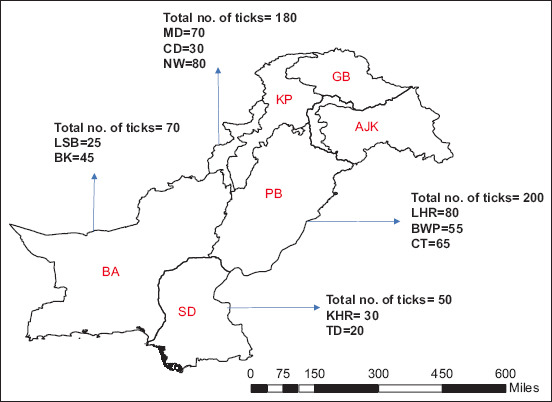
Map shows research areas from where tick samples were collected. This map was created by ArcGIS software version 10.3.1. KP=Khyber Pakhtunkhwa, BA=Balochistan, PB=Punjab, SD=Sindh, GB=Gilgit Baltistan, AJK=Jammu, and Kashmir, MD=Mardan, CD=Charsadda, NW=North Waziristan, LSB=Lasbela, BK=Barkhan, LHR=Lahore, BWP=Bahawalpur, CT=Cholistan, KHR=Karachi, TD=Tandojam.

Live engorged ticks were collected from different regions of Pakistan (Figure-1). A total of 500 ticks were collected from cattle using tissue forceps and placed in 50 mL Falcon tubes with perforated lids. All samples were transported in plastic zipper bags with small perforations to the Molecular Parasitology Laboratory at the UVAS, Lahore to allow further procedures.

### Morphological identification

**Figure-2:**
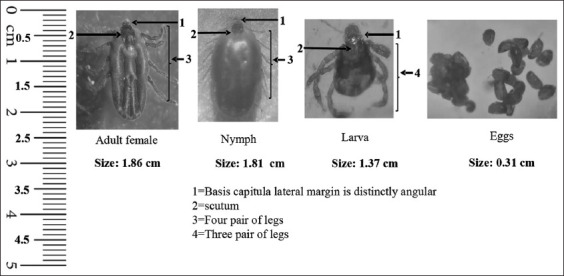
Morphological identification of *Rhipicephalus annulatus* tick and its stages.

All collected ticks were morphologically identified under a stereomicroscope using the tick electronic guide (Figure-2) [22]. Tick measurements and their stages were performed using ImageJ software (https://imagej.net/ij/download.html) [23]. In addition, ticks were sorted by species. Engorged female ticks were cleaned with sterile distilled water, dried with soft-tissue paper, and set aside for oviposition.

### Rearing of ticks

A few engorged female ticks were analyzed by PCR at the time of collection to confirm the intake of *A. marginale*. Infected and non-infected female ticks were placed separately in a biological oxygen demand (BOD) incubator at 27°C and 85% relative humidity. Non-infected larvae were cultured after hatching the eggs. After molting the nymphs from the larvae, they were fed on animals. After molting the female ticks from the nymphs, they were further cultured until engorgement. After oviposition, they were examined for the presence or absence of *A. marginale* when they died.

### Source of *A. marginale* and tick rearing on rabbits and calf

In our previous study, *A. marginale* was detected in the blood of infected crossbred Holstein cattle [24]. In the current study, *A. marginale* was further used for the inoculation of rabbits. Eight male rabbits of the *Oryctolagus cuniculus* breed and one crossbred Holstein cattle calf [25] were screened for *A. marginale* using PCR [26]. Rabbits and cattle calf were declared free from any type of infection by microscopic examination and PCR.

### Tick rearing on rabbits and calf

**Figure-3:**
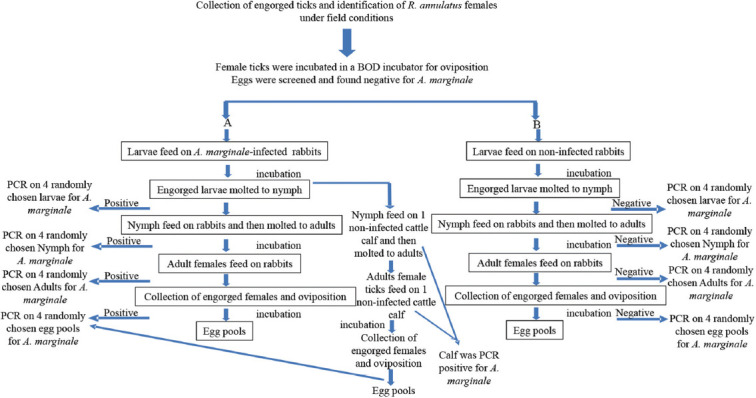
Flow chart of experiment: (A) Experimental group and (B) control group. Follow-up experiment for the screening of *Anaplasma marginale* from the ticks and blood of the rabbit and calf. BOD=Biological Oxygen Demand.

**Figure-4:**
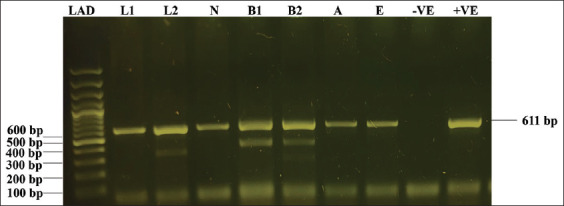
PCR detection of 16s rRNA gene of *A. marginale* in *Rhipicephalus annulatus* tick and rabbit and calf blood samples with specific primers of *A. marginale* (A) Lane LAD is the DNA Ladder of 100 bp (Gene Ruler 100 bp plus DNA ladder Ref: SM0323). Lane L1-L2 indicates positive samples of tick larvae, Lane N indicates positive samples of nymphs, Lane B1 indicates a positive sample of rabbit blood, Lane B indicates a positive sample of cattle calf blood, Lane A indicates a positive sample of adult tick, Lane E indicates a positive sample of tick eggs, Lane -VE indicates control negative, Lane +VE indicates control positive 611 bp of *A. marginale*. *A. marginale=Anaplasma marginale*.

Rabbits in Group A (4 rabbits) were inoculated with *A. marginale*-infected blood according to a previously described protocol [27, 28]. *R. annulatus* ticks free of *A. marginale* were reared on rabbits belonging to Group B (four rabbits) served as the control group, referred to as “clean rabbits.” One cattle calf was also used in this study. In Group A, 100 larvae were fed on rabbits infected with *A. marginale* on the 8^th^ day of infection. On the same day, 100 clean tick larvae were fed to the rabbits in Group B. The attached larvae were counted after 24 h of infestation, and the unattached larvae were removed. Larvae were allowed to feed on rabbits for a specified period of time, after which they were collected, counted, and stored in a BOD incubator. When larvae molt into nymphs, the nymphs fed on both rabbits and the calf. Engorged nymphs were collected 8–12 days later from the rabbits and 15 days later from the calf and allowed to molt into adults on both rabbits and the calf. Engorged female ticks were collected from the animals 21 days after molting to adults on rabbits and 25 days after molting to adults on the calf. The ticks were kept in the BOD incubator after feeding at every stage (Figure-3). A magnifying glass was used to count ticks attached to rabbits. Blood samples were collected from rabbits and calf before and after tick attachment. Blood and tick samples were analyzed by PCR to detect the presence of *A. marginale* (Table-1 and Figure-4). We determined the life cycle duration of female *R. annulatus* ticks attached to infected and non-infected rabbits. The durations before and during host attachment were preoviposition and oviposition, whereas the durations during host attachment were the larval, nymphal, and adult periods (Table-2).

**Table-1:** Infection rate in different stages of randomly chosen *R. annulatus* ticks after feeding on infected and non-infected groups of rabbits.

S. No.	Experimental Group (4 Rabbits inoculated with *A. marginale*)	PCR Results of *A. marginale* +Ve %	Experimental group (1 clean cattle calf)	Clean calf infected after rearing of nymphs	Control Group (Clean Rabbits)	PCR Results of *A. marginale* +Ve %
1	Larvae	4/4	Larvae	-	Larvae	0/4
2	Nymphs	4/4	Nymphs	-	Nymphs	0/4
3	Adults	4/4	Adults	1/1	Adults	0/4
4	Egg Pools	4/4	Egg Pools	2/2	Egg Pool	0/4

Sr. no: Serial Number; +Ve%: Positive percentagem, *A. marginale=Anaplasma marginale*

**Table-2:** The durations of the life cycle of infected and non-infected *R. annulatus* ticks fed on rabbits.

Biological parameters	Life cycle duration (Days) of *R. annulatus*					

	Non-infected ticks of rabbits			Infected ticks of rabbits		
	
	Range	Mean	Confidence interval 95%	Range	Mean	Confidence interval 95%
Preoviposition	2–9	4.2	3.611–4.833	1–9	4.4	2.282–6.468
Oviposition	15–22	17.6	16.99–18.40	15–21	18.5	16.55–20.45
Hatching	17–26	19.6	18.89–20.28	19–26	19.87	17.58–22.17
Free larval period	4–5	4.3	4.128–4.483	4–5	4.625	4.003–5.247
Larva	16–26	18.3	17.36–19.25	15–26	18.5	14.98–22.02
Nymph	2–9	5.3	4.729–5.994	2–9	5.5	3.357–7.643
Adult	6–9	7.47	7.154–7.791	6–10	8	6.736–9.264
Total life cycle	71–84	76.9	70.93–79.09	71–89	79.37	72.83–85.91

### DNA extraction

Genomic DNA was isolated from the blood samples of rabbits, female ticks, egg pools, larvae, and nymphs using the GeneJET Genomic DNA purification kit (Thermo Scientific, Van Allen Way, Carlsbad, California) according to the manufacturer’s protocol (https://assets.thermofisher.com/TFS-Assets/LSG/manuals/MAN0012663_GeneJET_Genomic_DNA_Purification_Kit_UG.pdf). For DNA extraction from blood samples, we thoroughly mixed 300 μL of blood with 20 μL of proteinase K and 400 μL of lysis buffer. subsequently, the mixture was incubated at 56°C in a water bath. Approximately 200 μL of absolute ethanol was then added and the mixture was thoroughly mixed. subsequently, the resulting mixture was transferred into a mini spin column and centrifuged at 6,000× *g* for 1 min. Next, 500 μL of wash buffer (I) was added, followed by buffer (II), and centrifugation at 12,000× *g* for 3 min. Finally, 200 μL of elution buffer was added and the mixture was centrifuged at 8,000× *g* for 1 min. DNA extraction from tick samples was initiated by crushing the tick samples with a sterile mortar and pestle. The triturated material was effectively mixed with 180 μL of digestion solution and 20 μL of proteinase K, followed by incubation at 56°C in a water bath. Post-incubation, 20 μL of RNase solution, 200 μL of lysis buffer, and 400 μL of 50% ethanol were added to the solution and thoroughly mixed. The resulting mixture was transferred into a mini spin column and centrifuged at 6,000× *g* for 1 min. subsequently, 500 μL of wash buffer (I) was added, buffer (II) was introduced and centrifuged at 12,000× *g* for 3 min. Finally, 200 μL of elution buffer was added and the mixture was centrifuged at 8,000× *g* for 1 min. DNA derived from this procedure was stored at –20°C until further analysis.

### PCR amplification

Detection of *A. marginale* in rabbit blood sample and tick stages through PCR was performed using G-STORM Thermocycler and Taq Polymerase (cat#k0171) with the primer pairs AF-5′-ATACCCTGGTAGTCCACGCT-3′ and AR-5′-GCAGTGTGTACAAGACCCGA-3′ specific for *A. marginale* [26]. A total volume of 25 µL was prepared for PCR. The reaction started with an initial denaturation at 95°C for 4 min, followed by 35 cycles of final denaturation at 95°C for 1 min, annealing at 50°C for 1 min, initial extension at 68°C for 1 min, and final extension at 68°C for 10 min. The amplified PCR products were analyzed on a 1.5% agarose gel after completion of PCR. A negative control of *A. marginale* [26] and a negative control (DEPC water: Diethyl pyrocarbonate) were included in the PCR run. The Gel/PCR Purification Mini Kit (WizPrep, Korea Ref no: W70150-300) was used to purify the PCR amplicons.

### Statistical analysis

Student’s t-test was performed using GraphPad prism version 6 software (https://graphpad-prism.software.informer.com/6.0/) to compare biological parameters of the life cycle of *R. annulatus* in Groups A and B.

## Results

### Analysis of tick identification

Five hundred ticks were collected from cattle in different provinces of Pakistan. The highest occurrence of *R. annulatus* ticks was recorded in Punjab (200/500, 40%), followed by Khyber Pakhtunkhwa (180/500, 36%), Balochistan (70/500, 14%), and Sindh (50/500, 10%). Lahore had the highest occurrence of ticks (80/200, 40%), followed by Cholistan (65/200, 32.5%) and Bahawalpur (55/200, 27.5%) in the Punjab region. In the Khyber Pakhtunkhwa region, North Waziristan had the highest occurrence of ticks (80/180, 44.4%), followed by Mardan (70/180, 38.5%) and Charsadda (30/180, 16.7%). Moreover, in Balochistan, Barkhan had the highest occurrence of ticks (45/70, 74.28%) and Lasbela (30/70, 42.8%) had the lowest occurrence. In the Sindh region, Karachi had the highest occurrence of ticks (30/50, 60%), whereas Tandojam had the lowest occurrence (20/50, 40%) (Figure-1).

Adult females (n = 280, 56%), nymphs (n = 160, 32%), and adult males (n = 60, 12%) were the most prevalent life stages of ticks. Only non-infected tick larvae were used for the entire experiment.

### PCR confirmation of *A. marginale*

To confirm the presence of *A. marginale* before and after the inoculation of infection, blood and tick samples were amplified using primers targeting the 16S rRNA gene. An amplicon of 611 base pairs was obtained in *Anaplasma*-positive samples and revealed the amplification of *A. marginale* in rabbits (before and after inoculation and infestation) and calf blood samples (before and after rearing of *A. marginale* exposed nymphs) as well as in *R. annulatus* larvae, nymphs, adults, and egg pools (Figure-4).

### Evidence of the transmission of *A. marginale* in *R. annulatus* ticks

Filial transmission of *A. marginale* was observed during the experiment in all randomly selected larvae, nymphs, and adult females collected from rabbits and a calf. However, there was no evidence of this transmission in clean ticks collected from rabbits. During the experiment, 40 larvae were attached. Four larvae were randomly selected from each rabbit and were found to be positive by PCR (4/4). subsequently, only 25 larvae that molted into nymphs and four nymphs originating from the same group of larvae on rabbits were randomly selected. All four tested positive by PCR (4/4). In addition, four nymphs originating from the same group of rabbit larvae were randomly selected for rearing on the calf and the calf tested positive by PCR. The positive controls consistently tested positive, whereas the negative controls did not show a band in all procedures. Furthermore, only 15 nymphs molted into adult ticks on rabbits and three molted into adult ticks on calf. Four adults from the first generation were randomly chosen from rabbits and one adult was randomly chosen from calf. All four adult ticks from rabbits and one adult tick from calf were found to be positive by PCR (4/4; 1/1, respectively) after laying eggs. When pools of eggs obtained from these females were tested, all four (4/4: 2/2, respectively) were also positive. PCR revealed that all tick stages reared on non-infected rabbits were negative. Transovarial, transstadial, and horizontal transmissions of *R. annulatus* ticks from experimental Group A were observed during the biological stages (Table-1 and Figures-3 and 4).

### Observation of biological stages of *R. annulatus*

Larvae, nymphs, and adults of *R. annulatus* were able to acquire *A. marginale* infection and maintain it by transstadial and transovarial transmission until the end of the experiment. Engorged females collected from infected rabbits were able to transmit *A. marginale* to their progeny. The life cycle of *R. annulatus* in infected and non-infected rabbits was determined. In infected and non-infected ticks, the complete life cycle had an average duration of 79.37 days (71–89 days) (95% Confidence interval [CI]: 72.83–85.91) and 76.9 days (71–84 days) (95% CI: 70.93–79.09), respectively. The difference in life cycle was non-significant, but it was slightly longer in ticks infected with *A. marginale* (Table-2).

## Discussion

In this study, *A. marginale* was transmitted horizontally, transstadially, and transovarially from *R. annulatus* tick stages through *in vivo* experimentation using rabbit as a model animal. Rabbits were inoculated with *A. marginale* infection and fed with *R. annulatus* larvae, nymphs, and then adult female ticks. *A. marginale* DNA was detected through PCR in the inoculated (infected) rabbits, exposed larvae, nymphs, adults, and egg pools for a complete life cycle of *R. annulatus*. *A. marginale* was also detected in calf blood samples after rearing of nymphs infected with *A. marginale*. In this study, we also reported the occurrence of *R. annulatus* in cattle across various provinces of Pakistan, revealing insights into the distribution of R. annulatus across different regions.

The results showed that Punjab exhibited the highest prevalence, with 40% of the collected ticks found in this province. Khyber Pakhtunkhwa recorded 36%, Balochistan 14%, and Sindh 10%, respectively. In this study, the highest occurrence of *R. annulatus* ticks was recorded in Punjab, which is consistent with earlier reports of *R. annulatus* occurrence in the same region [29]. On the other hand, the lowest occurrence of ticks was documented in Sindh, which is consistent with previous findings of ticks in Sindh region [30].

Transstadial or interstadial transmission of parasites occurs from the larval stage to the nymphal stage and then to the adult stage of ticks when feeding on an animal model. We confirmed 100% filial transmission of *A. marginale* within larvae, nymphs, and adult ticks through PCR, following the procedure documented in a previous study [31]. Scoles *et al*. [32] demonstrated transstadial transmission of *A. marginale* in *Rhipicephalus sanguineus* and *Dermacentor* *andersoni* feeding on cattle calves. Several studies have reported transstadial or interstadial transmission of *A. marginale* in *R. annulatus* [33] and *R. microplus* [9, 11], while feeding on cattle calves. To investigate transstadial transmission, we decided to use rabbits instead of cattle calves, which are more commonly used. This decision was influenced by previous studies that utilized rabbits as a model for culturing *Theileria annulata* [25] and *Babesia bigemina* [33], prompting us to select rabbits as our experimental model. The filial transmission is in agreement with other studies in which researchers found a 100% filial transmission rate similar to that of *Rickettsia rickettsii* in *Amblyomma aureolatum* [34], *R. sanguineus* [35], *D. andersoni* [36], and *Rickettsia amblyommii* in *Amblyomma auricularium* ticks [37].

Horizontal transmission refers to the spread of a parasite from the tick to the host and vice versa. In the case of a competent vector, horizontal transmission must be present in ticks [38, 39]. Horizontal transmission of *A. marginale* from larvae, nymphs, and adult female ticks was detected when these ticks and their life stages fed on clean rabbits (uninfected rabbits). Wolbachia is a rickettsial organism similar to *Anaplasma* that has been found in horizontal transmission through *R. annulatus* in cattle [40]. Males of one-host ticks such as *R. annulatus* or *R. microplus* readily move from one animal to another, enabling intrastadial transmission from infected cattle to non-infected cattle [33]. *A. marginale* can also transmit horizontally to uninfected cattle through unfed larvae descended from infected *R. microplus* ticks [13]. Horizontal transmission was achieved to clean rabbits by exposing them to unfed larvae, nymphs, and female ticks infected with *A. marginale* that had previously been fed on rabbits inoculated with *A. marginale* infection. Ticks show flexibility when choosing their hosts to adapt and survive. Although Ixodid ticks are generally not specific to a single host, they may develop a stronger preference for certain hosts over time [41], similar to the rabbits in our study.

Transovarial transmission is a vertical transmission of the parasite from a female tick to the larvae of the next-generation through the eggs [42]. Some studies have demonstrated the evidence of vertical transmission by finding the DNA of *A. marginale* through PCR in the eggs or unfed larvae of engorged female *R. microplus* [43] and *R. annulatus* [18] collected from infected cattle.

In this study, attempts to rear *R. annulatus* on infected (71–89 days with a mean value of 79.37 days) and clean (71–84 days with a mean value of 76.9 days) rabbits showed that larvae and nymphs succeeded in molting adults. Infected adult ticks fed on rabbits took a slightly longer time (+26.8 days) than non-infected ticks to complete their life cycle. This finding agrees with that reported by some researchers [44, 45], who reared *R. decoloratus* and *R. annulatus* ticks on non-infected rabbits. They found that *R. annulatus* and *R. decoloratus* larvae and nymphs molt on rabbits, whereas females fed slowly for 5 days without being partially engorged. We calculated the life cycle of *R. annulatus* on clean rabbits as 71–84 days with a mean of 76.9 days, whereas Abdel-Shafy (2018) calculated the life cycle as 59–82 days with a mean value of 70 days [44]. Six New Zealand white rabbits were used for rearing *R. annulatus*. In one group, larvae were allowed to feed until they became adult ticks. These adult ticks were then removed and fed again to different rabbits. In another group, the larvae were kept on the rabbits until fully fed females dropped off. Thus, the difference in days might be due to the difference in the incubation period observed off the rabbits. In our study, we observed a consistent decrease in tick numbers at each stage when rearing them on rabbits, similar to a previous study where deer mice were used for rearing *D. andersoni* ticks [46]. This decline can be attributed to various factors, such as ectoparasite density [47], host defenses, and host and ectoparasite ages [48].

## Conclusion

Using an *in vivo* approach on rabbits, the findings of our study demonstrate that transstadial, horizontal, and transovarial transmissions of *A. marginale* are feasible in *R. annulatus* ticks. In addition, we established a procedure for cultivating unfed *R. annulatus* ticks on a rabbit model that completes its entire life cycle. In future studies, rabbits can be used as a model for the evaluation of host-parasite interactions.

## Authors’ Contributions

MIR: Conceived and designed the study. SA: Collected the samples and analyzed the data. SA: Performed the experiments. SA and MIR: Analyzed and interpreted the data and drafted and revised the manuscript. ASA, KA, and JAK: Data analysis and validation. All authors have read, reviewed, and approved the final manuscript.
